# Beclin1 Deficiency Suppresses Epileptic Seizures

**DOI:** 10.3389/fnmol.2022.807671

**Published:** 2022-07-22

**Authors:** Min Yang, Peijia Lin, Wei Jing, Haokun Guo, Hongnian Chen, Yuanyuan Chen, Yi Guo, Yixue Gu, Miaoqing He, Junhong Wu, Xuejun Jiang, Zhen Zou, Xin Xu, Chengzhi Chen, Fei Xiao, Xuefeng Wang, Xin Tian

**Affiliations:** ^1^Chongqing Key Laboratory of Neurology, Department of Neurology, The First Affiliated Hospital of Chongqing Medical University, Chongqing, China; ^2^Center of Experimental Teaching for Public Health, Experimental Teaching and Management Center, Chongqing Medical University, Chongqing, China; ^3^Molecular Biology Laboratory of Respiratory Diseases, Institute of Life Sciences, Chongqing Medical University, Chongqing, China; ^4^Research Center for Environment and Human Health, School of Public Health, Chongqing Medical University, Chongqing, China; ^5^Department of Occupational and Environmental Health, School of Public Health and Management, Chongqing Medical University, Chongqing, China

**Keywords:** epilepsy, Beclin1, transgenic mice, excitatory synaptic transmission, dendritic spines

## Abstract

Epilepsy is a common disease of the nervous system. Autophagy is a degradation process involved in epilepsy, and in turn, seizures can activate autophagy. Beclin1 plays a critical role in autophagy and participates in numerous physiological and pathological processes. However, the mechanism underlying the effect of Beclin1 on epilepsy remains unclear. In this study, we detected increased expression of Beclin1 in brain tissues from patients with temporal lobe epilepsy (TLE). Heterozygous disruption of *beclin1* decreased susceptibility to epilepsy and suppressed seizure activity in two mouse epilepsy models. We further illustrated for the first time that heterozygous disruption of *beclin1* suppresses excitatory synaptic transmission, which may be caused by a decreased dendritic spine density. These findings suggest for the first time that the regulation of Beclin1 may serve as a strategy for antiepileptic therapy. In addition, Beclin1 participates in synaptic transmission, and the development of dendritic spines may be a biological function of Beclin1 independent of its role in autophagy.

## Introduction

Epilepsy is a common disease of the nervous system that is caused by the abnormal discharge of highly synchronized neurons; it is characterized by recurrent seizures (Fisher et al., 2014) and affects approximately 65 million people worldwide. Although more than 20 antiepileptic drugs (AEDs) have been developed and are used to treat epilepsy, approximately one-third of patients fail to achieve seizure control or soon become resistant to their effects (Pitkänen and Lukasiuk, 2011). Therefore, the identification of novel therapeutic targets and development of effective drugs that prevent or reverse the molecular mechanisms underlying epilepsy progression are urgently needed.

Based on accumulating evidence, autophagy may be involved in epilepsy (Egan et al., 2011). Autophagy is a process for degrading intracellular substances that is highly conserved among species and involves the transport of abnormal proteins, damaged organelles and other macromolecules to lysosomes for degradation (Levine and Kroemer, 2019). Some researchers have found that knockout of the autophagy gene ATG7 in mice leads to spontaneous epilepsy (Wong, 2013). Lafora disease, an autosomal recessive epilepsy syndrome, is caused mainly by deficiency of the phosphatase laforin or the ubiquitin ligase malin, and knockout of either enzyme results in the same clinical phenotype as defective autophagy (Criado et al., 2012). Hence, researchers have proposed that impaired autophagy might trigger the occurrence of epilepsy and conversely epilepsy might likewise result in the dysregulation of autophagy, which would further exacerbate epilepsy and create a vicious cycle (Li et al., 2018). However, the mechanism of autophagy in epilepsy has not been completely elucidated (McDaniel et al., 2011). Therefore, a deeper understanding of the mechanisms may be needed.

Beclin1 is a key molecule involved in the autophagy process that was first identified as a novel protein in 1998 (Liang et al., 1998). Subsequently, Beclin1 was confirmed to be a homolog of the yeast autophagy gene Apg6/Vps30, which compensates for the autophagy disorder caused by *apg6* gene defects. Therefore, *beclin1* was considered the first identified autophagy-related gene in humans (Liang et al., 1999). Beclin1 participates in many physiological and pathological processes by forming a complex with PI3K along with VPS34 and other proteins (Liang et al., 2006). Beclin1 is expressed in the nervous system and participates in a variety of neurodegenerative diseases, such as Alzheimer’s disease and Huntington’s disease (Ashkenazi et al., 2017). However, the potential role of Beclin1 in regulating epilepsy remains unclear. Therefore, in the present study, we aimed to investigate whether Beclin1 modulates epilepsy. We further observed whether the heterozygous disruption of *beclin1* affects neuronal synaptic transmission by performing whole-cell patch clamp recordings. We observed changes in the development of dendritic spines and autophagy in *beclin1*^±^ mice to elucidate the underlying mechanisms. These findings indicate that modulating Beclin1 may represent a new approach for preventing epilepsy and may provide new insights into the biological functions of Beclin1.

## Materials and Methods

### Human Brain Tissues

We obtained cerebral temporal lobe cortical tissues from patients with drug-refractory temporal lobe epilepsy (TLE) or patients with brain trauma who underwent surgery at The First Affiliated Hospital of Chongqing Medical University. According to the classification of epileptic seizures proposed by the International League Against Epilepsy (ILAE) in 2001 (Engel, 2001), patients diagnosed with TLE had typical epilepsy symptoms and electroencephalographic features and recurrent seizures despite having taken 3 or more different AEDs for more than 2 years. Age- and sex-matched patients who were treated for increased intracranial pressure secondary to traumatic brain injury and had no history of epilepsy, no exposure to AEDs, or no other history of neurological and psychiatric disorders were considered the control group. The surgery was performed using the anterior temporal lobe resection procedure (Falconer and Taylor, 1968), and the resected epileptic temporal lesion was localized using high-resolution magnetic resonance imaging, prolonged video-EEG monitoring, and/or positron-emission tomography (PET). The clinical features of the patients included in this study are summarized in Table 1.

**TABLE 1:** Clinical characteristics of patients with intractable TLE and control patients.

Number	Sex (M/F)	Age (Years)	Course (Years)	AEDs taken before surgery
T1	F	26	7	CBZ, VPA, OXC, TPM
T2	M	22	5	CBZ, TPM, CZP
T3	F	25	10	CBZ, PB, LTG, LEV
T4	M	24	8	VPA, CBZ, TPM, PB
T5	M	18	7	PHT, PB, CBZ, VPA
T6	F	35	10	CBZ, VPA, TPM, OCX
T7	F	22	9	CBZ, TPM, CZP
T8	M	29	14	CBZ, LTG, LEV, PB
C1	M	11	0	None
C2	F	38	0	None
C3	M	40	0	None
C4	F	34	0	None
C5	M	30	0	None
C6	F	17	0	None
C7	M	11	0	None
C8	M	25	0	None

*T, TLE temporal lobe epilepsy; C, control; M, male; F, female; CBZ, carbamazepine; LTG, lamotrigine; LEV, levetiracetam; OXC, oxcarbazepine; PHT, phenytoin; PB, phenobarbital; TPM, topiramate; VPA, valproic acid.*

### Animals

All experiments were conducted in accordance with the guidelines of the Guide for the Care and Use of Laboratory Animals. Healthy, specific pathogen-free (SPF) adult wild-type (WT) male C57BL/6J mice (weight 25 ± 2 g, age 8–10 weeks) were provided by the Experimental Animal Center of Chongqing Medical University. *beclin1*^±^ mice on a C57BL/6J background were obtained from the laboratory of Beth Levin as previously described (Liu and Wang, 2019). All mice were housed in groups of 5 mice per cage under standard conditions, including a 12-h light/dark cycle, temperature of 23 ± 1°C, relative humidity of 50 ± 10%, and an SPF environment with sufficient standard feed and water.

Epileptic brain tissues were obtained from mice with kainic acid (KA)-induced epilepsy presenting spontaneous recurrent seizures (SRSs) or mice that were fully kindled after pentylenetetrazol (PTZ) treatment. Correspondingly, control brain tissues were obtained from mice injected with saline under the same conditions.

### Pentylenetetrazol - Kindled Epilepsy Model

Mice received intraperitoneal injections of 35 mg/kg PTZ every other day for 30 days to establish the PTZ-kindled chronic epilepsy model. After each injection, the seizures experienced the mice in each group were graded for 30 min according to the Racine scale (Racine, 1972): grade I, clustered whisker and facial movements with chewing motions; grade II, facial spasm with rhythmic nodding; grade III, unilateral forelimb clonus or tonus; grade IV, bilateral forelimb tonic–clonic seizures with rearing; and grade V, loss of posture or generalized tonic–clonic seizures (GTCSs) with a fall or death.

### Establishment of the Kainic Acid -Induced Chronic Epilepsy Model and Local Field Potential Recording

To establish the KA-induced chronic epilepsy model, mice were anesthetized and fixed on a stereotaxic apparatus. Then, 1.0 nmol of KA (Sigma–Aldrich) in 50 ml of saline was injected into the hippocampi [anteroposterior (AP), −1.6 mm; mediolateral (ML), −1.5 mm; dorsoventral (DV), −1.5 mm] of the mice. Diazepam (10 mg/kg) was administered to terminate non-convulsive status epilepticus (SE) 2 h after the injection. The mice were continuously recorded with a digital video camera for 1 month. The observers counted the number of grade IV or V SRSs in each group. The latency and total number of SRSs were analyzed.

After video monitoring of KA-induced chronic seizures, intracranial local field potential (LFP) recordings were performed as previously described (Yang et al., 2018). Two stainless steel screws were implanted in the anterior cranium, and a platinum-iridium alloy microwire (25 μm in diameter; Plexon, Hong Kong, SAR, China) was implanted into the right dorsal hippocampus (AP, 1.6 mm; ML, 1.6 mm; DV, 1.5 mm). The guide cannula, the microwire, and a U-shaped frame were cemented to the skull to hold the head. Each mouse was continuously recorded for 30 min using a MAP data acquisition system (Plexon, Dallas, TX, United States). The LFP data were analyzed using NeuroExplorer (Nex Technologies, Littleton, MA, United States). Each mouse was continuously recorded for 30 min to assess LFP signals. A cluster of paroxysmal discharges with amplitudes two times greater than those at baseline that occurred spontaneously with a frequency greater than 1 Hz and a duration greater than 5 was recorded as a seizure-like event (SLE). The total number of SLEs, the duration of each SLE and the spectrograms obtained during the 30-min recording were analyzed using NeuroExplorer VR software (Version 4, Plexon, Hong Kong SAR, China).

### Protein Extraction and Western Blot Analysis

For protein extraction, mice were anesthetized with 1% sodium pentobarbital, and the cortex and hippocampus were isolated from the brain of each mouse and placed on ice. A RIPA protein extraction kit (P0013B, Beyotime Biotechnology, China) containing phenylmethylsulfonyl fluoride (PMSF) was used to extract total protein. An enhanced bicinchoninic acid (BCA) protein assay kit (P0012S, Beyotime Biotechnology, China) was used to determine the protein concentrations.

Western blotting was completed using published protocols (Li et al., 2016). SDS–PAGE Sample Loading Buffer-5 × (P0012A, Beyotime Biotechnology, China) was used to denature the proteins. Extracts were resolved on SDS–PAGE gels (5% spacer gel and 10% separating gel) and then subjected to western blot analysis. The following antibodies were used in the present study: rabbit anti-Beclin1 antibody (11306-1-AP, Proteintech, China, RRID:AB_2259061), rabbit anti-LC3 antibody (14600-1-AP, Proteintech, China, RRID:AB_2137737), rabbit anti-p62/sequestosome-1 (SQSTM1) antibody (18420-1-AP, Proteintech, China, RRID:AB_10694431), rabbit anti-GAPDH antibody (10494-1-AP, Proteintech, China, RRID:AB_2263076), and goat anti-rabbit IgG antibody (SA00001-2, Proteintech, China, RRID:AB_2722564). The bands were visualized using Western Bright ECL reagent (Advansta, United States) and a Fusion FX5 image analysis system (Vilber Lourmat, France).

### Primary Hippocampal Neuron Culture, Plasmid Transfection and Drug Intervention

Primary neuronal cultures were established as previously described (Guo et al., 2020). Postnatal brain tissue was dissected from early postnatal mice, and the tissues were digested with trypsin and mechanical dissociation to obtain neurons. The cell suspension was diluted with DMEM supplemented with 20% FBS (Gibco, Thermo Fisher Scientific). Neurons were plated at a density of 100,000 cells on poly-L-lysine–coated 35-mm dishes or glass coverslips in 6-well plates and incubated in a cell culture incubator at 37°C for 4 h. Four hours after plating, the cells were maintained in Neurobasal medium supplemented with B27, 2 mM L-glutamine, 100 U/ml penicillin, and 100 μg/ml streptomycin (Invitrogen). Neurons were transfected with a plasmid encoding green fluorescent protein (GFP) using the calcium phosphate transfection method at 7 days (DIV7) *in vitro* to visualize their spines. Certain cells were treated with 10 μM 3-methyladenine (3-MA) or 100 nM rapamycin (RAPA) (Selleck, United States) to assess the effects of drug interventions.

### Immunofluorescence Staining and Semiquantitative Analysis

For the preparation of brain tissue sections, male C57BL/6 mice (7–8 weeks old) were anesthetized with 1% sodium pentobarbital and successively subjected to ischemia–reperfusion with 40 ml of saline and 40 ml of 4% paraformaldehyde. Brain tissues were removed, soaked in 4% paraformaldehyde for 12 h and then soaked in a 30% sucrose solution for 48 h. The tissues were embedded in optimum cutting temperature (OCT) compound (Sakura, 4583) and cut into 15 μm sections with a cryotome (Leica CM1950).

For IF staining, the tissue sections were permeabilized with 0.4% Triton X-100 at 37°C for 30 min, soaked in a sodium citrate solution (P0086, Beyotime, China), heated in a microwave oven for 3 min at high temperature and 10 min at low temperature, and then blocked with the working dilution of goat serum (C0265, Beyotime, China) for 1 h at 37°C. The sections were then incubated overnight with a mixture of primary antibodies at 4°C followed by washing and incubated with secondary antibodies in the dark at room temperature (RT) for 1 h for surface staining. For immunostaining of cultured neurons, neurons were fixed with 4% paraformaldehyde/4% sucrose in PBS for 30 min at RT, permeabilized with 0.3% Triton X-100 for 15 min and blocked with 10% goat serum for 30 min at RT. Neurons were incubated with the primary antibody at 4°C for 6 h and incubated with the secondary antibody at RT for 1 h. Images were acquired with a confocal microscope (ZEISS, Wetzlar, Germany).

The primary antibodies applied included a rabbit anti-Beclin1 antibody (11306-1-AP; Proteintech, China, RRID:AB_2259061), mouse anti-PSD-95 antibody (MAB1596, Millipore Sigma, United States, RRID:AB_2092365), and guinea pig anti-vGluT1 antibody (135304, Synaptic Systems, Germany, RRID:AB_887878). The fluorophore-conjugated secondary antibodies used were goat anti-guinea pig Alexa Fluor 647 (ab150187; Abcam, Britain, RRID:AB_2827756), goat anti-rabbit Alexa Fluor 488 (A-11008; Invitrogen, United States, RRID:AB_143165), goat anti-mouse Alexa Fluor 549 antibodies (A-11005, Invitrogen, United States, RRID:AB_141372), goat anti-rabbit Alexa Fluor 594 antibodies (A23420, Abbkine, United States), and goat anti-mouse Alexa Fluor 488 antibodies (A23210, Abbkine, United States).

For the semiquantitative analysis, staining was performed under the same experimental conditions, and the images were captured with the same laser confocal excitation light intensity. Analyses of the fluorescence intensity of Beclin1 puncta and colocalization were performed using Image-Pro Plus 6.0 software. The dendritic spine density was analyzed using ImageJ software. Puncta positive for pre- and postsynaptic terminals were counted in 10 μm segments along the length of the dendrite.

### Cell Viability Assay

Cell viability was measured by MTT Kit (Meilunbio, China) according to the manufacturer’s protocol. Neurons were seeded onto 96-well plates at a density of 2 × 10^3^ cells/well and cultured for 24 h. Then, the cells were treated with MTT reagent for 4 h. Formazan solution was incubated with the cells for 4 h at 37°C. A multifunction enzyme-linked analyzer (Varioskan LUX, Thermo Fisher Scientific, United States) was used to detect the absorbance value (OD) at 570 nm.

### Nissl Staining

Frozen tissue sections were prepared for staining. Sections from mice in each group were washed with water, immersed in Nissl staining solution (Beyotime, China) for 30 min, and washed with water 2 times. Then, the tissue was dehydrated with ethanol and washed with xylene. Sections were analyzed using a brightfield microscope (Germany, ZEISS).

### Golgi–Cox Staining

An FD Rapid Golgi-Stain Kit (FD Neuro-technologies, Ellicott City, MD, United States) was used to perform Golgi–Cox staining according to the manufacturer’s instructions. Mice were anesthetized with 1% sodium pentobarbital, and their brain tissues were removed quickly. The tissues were immersed in Golgi–Cox solutions A and B for 2 weeks in the dark at RT, transferred to solution C and then incubated for 72 h at RT. Slices (150 μm thick) were cut using a vibratome. For Golgi–Cox staining, the sections were mounted on 3% gelatin-coated glass slides, air-dried, stained with solutions D and E, dehydrated in alcohol, cleared with xylene and mounted using resinous medium. Dendritic spines were imaged using a 20× or 40× objective and captured with a ZEISS digital camera (Wetzlar, Germany). For the quantification of the dendritic spine density, dendritic segments in layer III of the cortex were randomly selected, and counting was performed by an experimenter who was blinded to the group of each sample.

### Whole-Cell Patch-Clamp Recordings

Whole-cell patch-clamp recordings were performed as previously described (Zhang et al., 2019). Mice were anesthetized, and 300-μm brain slices were prepared (Leica, Germany, VP1200S Vibratome). The slices were prepared in an ice-cold solution containing 60 mM NaCl, 100 mM sucrose, 2.5 mM KCl, 1.25 mM NaH_2_PO_4_•2H_2_O, 20 mM D-glucose, 26 mM NaHCO_3_, 1 mM CaCl_2_, and 5 mM MgCl_2_•6H_2_O saturated with 95% O_2_ and 5% CO_2_. A storage chamber containing Mg^2+^-free artificial cerebrospinal fluid [ACSF; 125 mM NaCl, 2.5 mM KCl, 2 mM CaCl_2_, 26 mM NaHCO_3_, 1.25 mM KH_2_PO_4_, and 25 mM glucose (pH 7.4) bubbled with 95% O_2_/5% CO_2_] was used for slice recovery. The slices were fully submerged in the same flowing Mg^2+^-free ACSF (4 ml/min) at RT for recording.

A depolarizing current of 500 ms in the current clamp mode starting from −50 pA and increasing at increments of 20 pA was used to induce action potentials (APs) to explore the intrinsic excitability of neurons. The first current step that was able to induce AP firing in a neuron was regarded as the rheobase. The internal solution contained the following components: 60 mM K_2_SO_4_, 60 mM *N*-methyl-D-glucamine, 40 mM HEPES, 4 mM MgCl_2_ •6H_2_O, 0.5 mM BAPTA, 12 mM phosphocreatine, 2 mM Na_2_ATP, and 0.2 mM Na_3_GTP.

Glass pipette electrodes were filled with the following internal solution to measure the miniature inhibitory postsynaptic currents (mIPSCs): 100 mM CsCl, 10 mM HEPES, 1 mM MgCl_2_, 1 mM EGTA, 5 mM MgATP, 0.5 mM Na_3_GTP, 12 mM phosphocreatine, and 30 mM *N*-methyl-D-glucamine (NMG) (pH 7.4, 280 to 290 mOsm). The membrane potential was held at −70 mV in voltage-clamp mode, and mIPSCs were recorded in the presence of 20 μM 6,7-dinitroquinoxaline-2,3 (1H,4H)-dione (DNQX), 50 μM dl-2-amino-5-phosphonovaleric acid (D-APV), and 1 μM tetrodotoxin (TTX). In addition, glass pipette electrodes were filled with an internal solution containing 130 mM CsMeSO_4_, 10 mM CsCl_2_, 10 mM HEPES, 4 mM NaCl, 1 mM MgCl_2_, 1 mM EGTA, 5 mM MgATP, 0.5 mM Na_3_GTP, 12 mM phosphocreatine, and 5 mM NMG (pH 7.4, 280 to 290 mOsm) to record mEPSCs. mEPSCs were recorded at a holding potential of −70 mV in the presence of 1 μM TTX and 100 μM picrotoxin (PTX).

Evoked currents were recorded to evaluate NMDAR- and AMPAR-mediated EPSCs. The glass microelectrodes were filled with the same solution used to record the mEPSCs. A bipolar stimulation electrode located approximately 50 μm rostral to the recording electrode in the same layer was used to evoke AMPAR- and NMDAR-mediated synaptic responses. In the presence of 100 μM PTX, at −70 mV, the peak amplitude of the evoked EPSCs was identified as the AMPAR-mediated current; at + 40 mV, the amplitude of the evoked EPSCs at 50 ms post-stimulus was identified as the NMDAR-mediated current.

For the analysis of the paired-pulse ratio (PPR), the holding potential was −70 mV in the presence of 100 μM PTX. The interval for paired stimulations was set at 50 ms. The PPRs were calculated as the ratio of the second peak amplitude to the first peak amplitude.

The signals were acquired with a MultiClamp 700B amplifier (Axon, United States), followed by recording using pClamp 9.2 software (Molecular Devices, Sunnyvale, CA, United States). Synaptic activity was analyzed using the Mini Analysis Program (Synaptosoft, Leonia, NJ, United States) and pClamp 9.2 software (Molecular Devices, Sunnyvale, CA, United States).

### Statistical Analysis

Statistical analyses were performed using Prism 6.0 software (GraphPad, San Diego, CA, United States). Unpaired two-tailed Student’s *t*-test or one-way analysis of variance (ANOVA) was used for analyses. Group differences in the mean seizure score were evaluated with repeated-measures ANOVA. For the electrophysiological tests, the n values represent the numbers of neurons and slices. All the results are presented as the means ± SEMs, and *p* < 0.05 was considered to indicate statistical significance.

## Results

### Beclin1 Expression Was Increased in Patients With Temporal Lobe Epilepsy and Epileptic Mice

**FIGURE 1:**
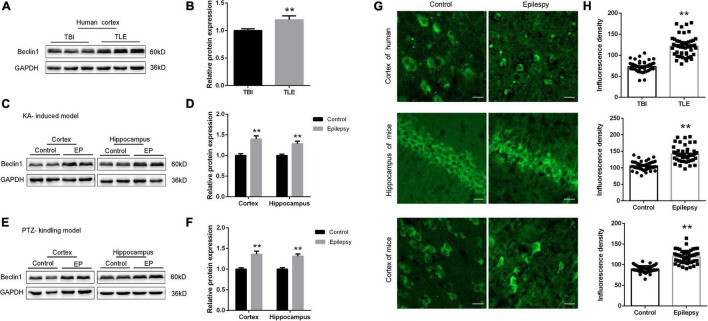
Beclin1 expression was increased in patients with TLE and epileptic mice. **(A,B)** Beclin1 protein levels in patients with traumatic brain injury (TBI) and patients with epilepsy (*n* = 8 patients per group). **(C,D)** The Beclin1 protein levels in cortex and hippocampus from wild-type (WT) mice and kainic acid (KA)-treated mice (*n* = 8 mice per group). **(E,F)** The Beclin1 protein levels in cortex and hippocampus from WT mice and pentylenetetrazol (PTZ)-treated mice (*n* = 8 mice per group). **(G,H)** IF staining and semi-quantitative analysis of Beclin1 puncta fluorescence intensity in cortical and hippocampal tissues from patients with temporal lobe epilepsy (TLE) and two epileptic mouse models (*n* = 50 samples per group). Scale bars: 50 μm. Data are presented as the means ± SEM, **p < 0.01. Student’s *t*-tests were performed.

Beclin1 has been shown to reflect change in autophagy under conditions of epilepsy (Li et al., 2018). We measured Beclin1 protein levels in temporal cortical tissues obtained from patients with TLE and non-epileptic patients using western blot analysis to verify the relationship between Beclin1 and epilepsy. Beclin1 expression in the temporal cortex was higher in patients with TLE than in non-epileptic patients (Figures 1A,B), and a similar result was observed in the cortical tissues obtained from the KA-induced (Figures 1C,D) and PTZ-kindled model mice (Figures 1E,F). The hippocampus is an area of important epileptic pathological changes and plays a key role in the occurrence and development of epilepsy. We next measured the Beclin1 protein level in hippocampal tissues from KA- and PTA-induced epileptic animal models. Compared with control tissues, Beclin1 expression was also increased in the epileptic tissue (Figures 1D,F). IF staining and a semiquantitative analysis were also performed (Figure 1G). The fluorescence density of Beclin1 puncta was higher in both the cortex and hippocampus of the epilepsy group than in the control group (Figure 1H), that was consistent with the results of the western blot analysis.

### Knockdown of *beclin1* Altered Autophagy

**FIGURE 2:**
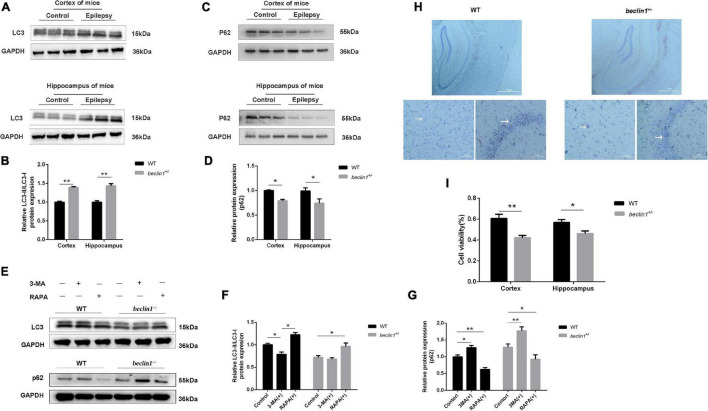
Autophagy was activated in epilepsy but suppressed in *beclin1*^±^ mice. **(A,B)** LC3-II/LC3-I protein levels in the cortex and hippocampus of brain tissues from wild-type (WT) and epilepsy model mice (*n* = 3 mice per group). **(C,D)** p62 protein levels in the cortex and hippocampus of brain tissues from WT and epilepsy model mice (*n* = 3 mice per group). **(E,G)** LC3-II/LC3-I and p62 protein level in cultured primary neurons from WT and *beclin1*^±^ mice under basal conditions and upon autophagy induction (RAPA)/blockade (3-MA) (*n* = 3 samples per group). **(H)** Nissl staining of WT and *beclin1*^±^ mouse brain slices. Scale bars: 52 μm. **(I)** The viability of cultured neurons dissected from WT and *beclin1*^±^ mice was detected using the MTT assay (*n* = 6 samples per group). Data are presented as the means ± SEM. *p < 0.05 and **p < 0.01. Student’s *t*-tests and one-way ANOVA were performed.

The dynamic process of autophagy consists of two aspects: the formation and degradation of autophagosomes. Microtubule-associated protein light chain 3 (LC3) is a marker of autophagy, and the LC3-II/LC3-I ratio correlates with the number of autophagosomes (Mizushima and Yoshimori, 2007). In the present study, we measured the protein levels of LC3 (Figure 2A) and adaptor protein p62 (SQSTM1), a substrate protein of autophagy (Figure 2C). Among epileptic mice, the LC3-II/LC3-I ratio was increased (Figure 2B), and the SQSTM1/p62 level was decreased compared with those in the WT mice (Figure 2D). In addition, we also detected the expression of LC3 and p62 in cultured neurons (Figure 2E). As a result, the decreased LC3 level observed in *beclin1*^±^ mice was increased by the autophagy inducer RAPA, but the autophagy blocker 3-MA did not further reduce the expression of LC3 (Figure 2F). However, increased p62 levels were decreased by RAPA and further increased by 3-MA (Figure 2G). Because the autophagy impairment in *beclin1*^±^ mice might result in tumorigenesis and/or neuronal loss, Nissl bodies in nerve cells were identified using Nissl staining. In the WT mouse, hippocampal structures were clear, nerve cells were arranged neatly, and the cytoplasm was rich in Nissl bodies. In *beclin1*^±^ mice, hippocampal structures were chaotic, nerve cells were sparsely arranged, and Nissl bodies were decreased (Figure 2H). In addition, we determined cell viability using MTT assays. The viability of cultured cortical and hippocampal neurons from *beclin1*^±^ mice was decreased compared to that in neurons from WT mice (Figure 2I).

### Knockdown of *beclin1* Decreased Epileptic Activity in the Kainic Acid -Induced Epilepsy Model

Altered Beclin1 expression may be an epiphenomenon or indicate that Beclin1 plays a causal role in epilepsy. We subsequently performed behavioral experiments to investigate whether incomplete knockout of *beclin1* affected seizure activities and epileptiform discharges in two epilepsy models.

**FIGURE 3:**
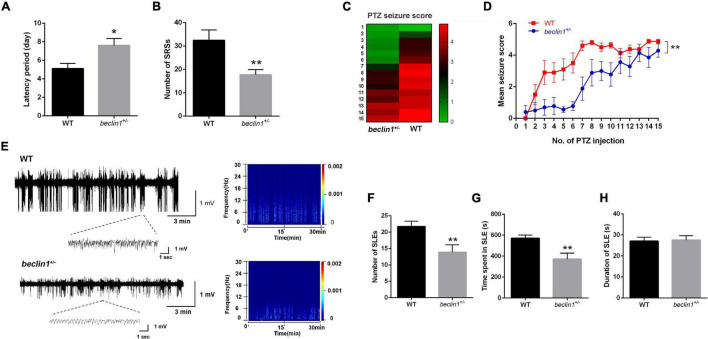
Knockdown of *beclin1* decreased seizure susceptibility and epileptic activity. **(A,B)** Quantitative analysis of the spontaneous recurrent seizure (SRS) latency and total number of SRSs in the kainic acid (KA)-induced epilepsy model in the two groups (*n* = 10 mice per group). **(C,D)** Mean seizure scores recorded after the injection of pentylenetetrazol (PTZ) in the two groups on alternate days (*n* = 10 mice per group). **(E)** Representative traces of local field potentials (LFPs) from the wild-type (WT) and *beclin1*^±^ groups. **(F–H)** Quantitative analysis of the number, time spent, and duration of seizure-like event (SLE) of the LFPs from two groups (*n* = 8 mice per group). Data are presented as the means ± SEM. *p < 0.05 and **p < 0.01. Group differences in the mean seizure score during PTZ kindling were evaluated with repeated-measures ANOVA. Student’s *t*-tests were performed.

In mice with KA-induced epilepsy, we counted only grade IV and V seizures, since grade I-III seizures were easily overlooked or were ambiguous. The first SRSs in the *beclin1*^±^ group appeared at approximately 8 days on average, but appeared on day 5 in the WT group (Figure 3A). In addition, the average total number of SRSs within 30 days was 18 in the *beclin1*^±^ group and approximately 32 in the WT group (Figure 3B). Thus, the heterozygous disruption of *beclin1* prolonged the latency of SRSs and decreased the total number of SRSs in the KA-induced epilepsy model.

### Knockdown of *beclin1* Decreased Seizure Susceptibility in the Pentylenetetrazol - Kindled Epilepsy Model

We selected another chronic epilepsy model with different mechanisms to avoid limiting our study to a single model. In the PTZ-kindled mouse model, the mice in the WT group exhibited grade III seizures after the administration of subthreshold doses of PTZ, and grade V seizures began to appear after the administration of the 8th–9th doses (Figures 3C,D). In contrast, in the *beclin1*^±^ group, grade III seizures began to appear at the 8th–9th PTZ injection, but no grade V seizures were observed after 15 injections (Figures 3C,D). Based on these results, the mice in the *beclin1*^±^ group had lower seizure scores and decreased seizure susceptibility than those in the WT group.

### Beclin1 Affected Local Field Potentials in the Hippocampi of Epileptic Mice

The judgment of epileptic seizures based on behavior has some limitations, since some subclinical epileptic discharges may be characterized only by abnormal EEGs. A more convincing judgment might be obtained by recording abnormal electrophysiological epileptiform discharges and SLEs during seizure intervals than by observing behavior. Therefore, after the behavioral analysis in the KA model, we obtained stable LFPs from each mouse continuously for 30 min, and SLEs were recorded in the two groups (Figure 3E). The total number of SLEs, the duration of each SLE, and the time spent in all SLEs were analyzed within the recorded 30 min. The total number of SLEs in the *beclin1*^±^ group was significantly lower than that in the WT group (Figure 3F). Interestingly, the total time spent in SLEs was significantly reduced in *beclin1*^±^ group (Figure 3G), but the duration of a single SLE of two groups was not significantly different (Figure 3H). These results suggest that the heterozygous disruption of *beclin1* affected SLEs in the interictal phase in the KA-induced epilepsy model by decreasing the number of SLEs and reducing the total time spent of SLEs but not by affecting the duration of a single SLE.

### Beclin1 Altered Excitatory Synaptic Transmission in Brain Slices

Intrinsic excitability or altered synaptic transmission may increase neuronal firing. We subsequently performed whole-cell patch-clamp recordings of hippocampal CA1 pyramidal neurons in mouse brain slices. First, the intrinsic excitability of the neurons was assessed, and no difference was observed between the WT and *beclin1*^±^ groups (Supplementary Figures 1C–G); therefore, we speculated that *beclin1*^±^ might affect synaptic transmission.

**FIGURE 4:**
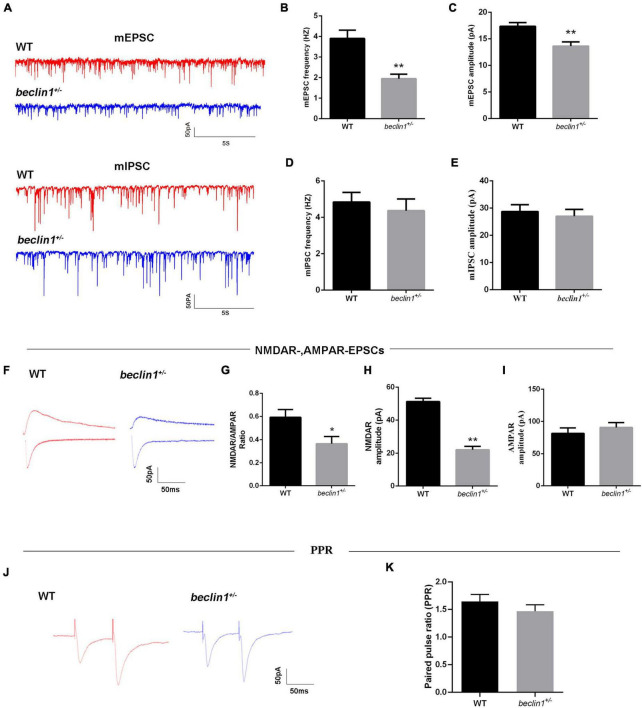
Knockdown of *beclin1* affected excitatory synaptic transmission. **(A)** Representative traces of mEPSCs and miniature inhibitory postsynaptic currents (mIPSCs) from the two groups. **(B,C)** Quantitative analysis of the frequency and amplitude of the mEPSCs [wild-type (WT) group: *n* = 10, *beclin1*^±^ group: *n* = 8]. **(D,E)** Quantitative analysis of the frequency and amplitude of the mIPSCs (*n* = 12 per group). **(F)** Representative traces of NMDAR-mediated and AMPAR-mediated EPSCs from the two groups. **(G–I)** Summary of the EPSC amplitude from the two groups (*n* = 6 per group). **(J)** Representative traces of the paired-pulse ratios (PPRs) for AMPA-mediated EPSCs at three different interstimulus intervals. **(K)** Summary of the PPRs between the two groups (*n* = 6 per group). Data are presented as the means ± SEM. *p < 0.05 and **p < 0.01. Student’s *t*-tests were performed.

**FIGURE 5:**
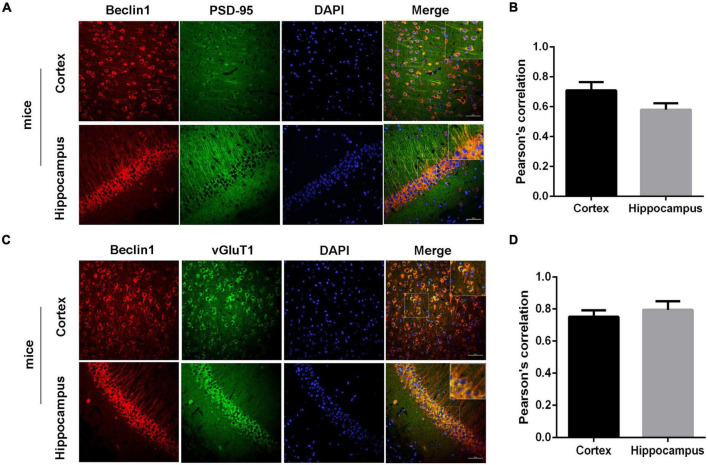
Beclin1 was colocalized with an excitatory synaptic protein. **(A)** Representative image showing the distributions of Beclin1 and the excitatory postsynaptic protein PSD-95 in mouse brain sections. **(B)** The extent of the colocalization was quantified using Image-Pro Plus software. **(C)** Representative image showing the distributions of Beclin1 and the excitatory presynaptic protein vGluT1 in mouse brain sections. **(D)** The extent of colocalization was quantified [wild-type (WT) group: *n* = 11, *beclin1*^±^ group: *n* = 9]. Data are presented as the means ± SEM. Scale bars: 50 μm. Student’s *t*-tests were performed.

Then, we recorded mEPSCs and mIPSCs in neurons of the brain slices (Figure 4A). Compared with those of the WT group, both the amplitude and frequency of mEPSCs were decreased in the *beclin1*^±^ group (Figures 4B,C), but significant differences in mIPSCs were not observed between the two groups (Figures 4D,E). Thus, *beclin1* knockdown mainly affected excitatory synaptic transmission. We also identified the subcellular location of Beclin1 in mouse brain tissues to investigate this hypothesis. We immunostained neurons with antibodies against Beclin1 combined with antibodies against the excitatory postsynaptic membrane protein PSD-95 and the excitatory presynaptic membrane protein vGluT1 (Figure 5A) and vGluT1 (Figure 5C). The colocalization analysis suggested that Beclin1 was significantly correlated with PSD95 (Figure 5B) and vGluT1 (Figure 5D).

We then measured evoked EPSCs to investigate whether the altered excitatory synaptic transmission was caused by AMPAR-mediated or NMDAR-mediated currents (Figure 4F). We observed decreases in the NMDAR/AMPAR ratio (Figure 4G) and in the average amplitude of the NMDAR-mediated EPSCs (Figure 4H) from the *beclin1*^±^ mice. However, AMPAR-mediated EPSCs were unchanged between the groups (Figure 4I). Alterations in NMDAR-mediated synaptic responses may occur presynaptically or postsynaptically. Therefore, the paired pulse ratios (PPRs) were recorded (Figure 4J), and no significant difference was observed between the two groups (Figure 4K). Taken together, these results indicate that *beclin1* may modulate glutamatergic transmission through a postsynaptic mechanism rather than a presynaptic mechanism.

### The Dendritic Spine Density Was Decreased in *beclin1^±^* Mice

**FIGURE 6:**
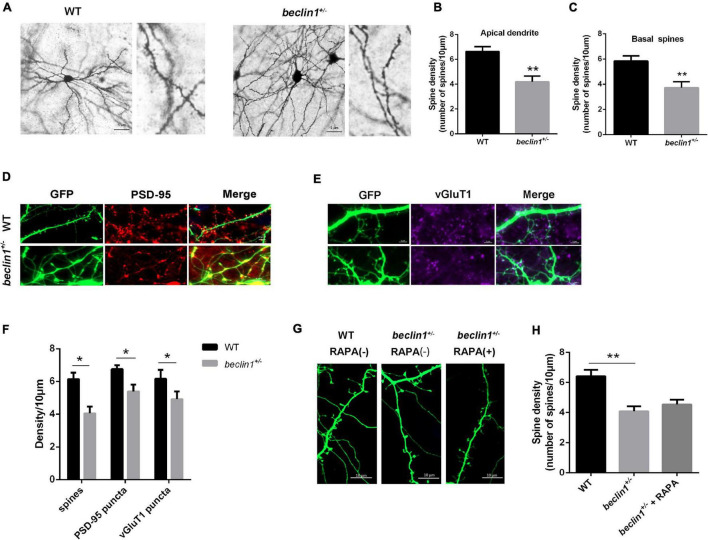
Knockdown of *beclin1* altered the dendritic spine density. **(A)** Representative image of the apical dendritic spine morphology of pyramidal neurons in brain slices from wild-type (WT) and *beclin1*^±^ mice detected using Golgi staining. Scale bars: 5 μm. **(B,C)** Quantification of the apical and basal dendritic spine density in the two groups (*n* = 5 mice/genotype, 21 cells from WT mice and 16 cells from *beclin1*^±^). **(D,E)** Representative image of dendrites and PSD-95/vGluT1 staining in cultured neurons from the two groups. **(F)** Quantification of the dendritic spine density and PSD-95/vGluT1 puncta density in the two groups (*n* = 12 cells). **(G,H)** Representative image and quantification of the dendritic spine density in cultured neurons from WT and *beclin1*^±^ mice under basal conditions and upon autophagy induction (RAPA) (*n* = 3 mice/genotype, 15 neurons per group). Spines on apical dendrites in the stratum radiatum (SR) located within 20 μm from the soma were counted. Data are presented as the means ± SEM. *p < 0.05 and **p < 0.01. Student’s *t*-tests and one-way ANOVA were performed.

Postsynaptic activity is closely related to the morphology of dendritic spines. We then examined whether Beclin1 was involved in dendritic morphogenesis in brain slices stained with a Golgi kit (Figure 6A). As a result, both apical and basal dendritic spine densities of cortical pyramidal neurons were lower in the *beclin1*^±^ group than in the WT group (Figures 6B,C). A similar trend was observed in cultured hippocampal neurons (Figures 6E,H). In addition, the presynaptic and postsynaptic regions were labeled with antibodies against PSD-95 (Figure 6D) and vGluT1, respectively (Figure 6E), to quantify whether changes in synaptic contacts accompanied the decreased dendritic spine density, and the results suggested that the densities of vGluT1 and PSD-95 puncta were both decreased in the *beclin1*^±^ group (Figure 6F).

As *beclin1* knockdown caused autophagy defects, we treated cultured neurons from *beclin1*^±^ mice with the autophagy inducer RAPA to further investigate whether the changes in the dendritic spines were caused by impaired autophagy (Figure 6G). However, no significant difference was observed in the dendritic spine density between the two groups (Figure 6H).

Prior to using the primary cultured neurons in this experiment, Beclin1 expression was confirmed by western blotting (Supplementary Figure 1A). Next, *beclin1*^±^ mice and WT mice were identified, and the expression of the Beclin1 protein in the *beclin1*^±^ group was significantly lower than that in the WT group (Supplementary Figure 1B). This finding indicated that Beclin1 protein expression was reduced, as expected, in the cortex and hippocampus of *beclin1*^±^ mice.

## Discussion

Numerous studies have focused on the underlying pathophysiological mechanisms of epilepsy, and the proposed mechanisms involve proteins, channels, receptors, signaling pathways and enzymes (Zavala-Tecuapetla et al., 2020). The role of autophagy in the development of epilepsy has received increasing attention, as defective autophagy has been shown to induce epilepsy (Wong, 2013). Thus, we investigated the role of Beclin1, a core molecule in autophagosome formation, in the occurrence and development of epilepsy.

Epileptogenesis is the process by which abnormal electrical activity develops into chronic epilepsy after functional changes occur in normal brain tissue. During this period, neurons and non-neuronal cells in brain tissue change at different levels, including the genetic, epigenetic, molecular and structural levels (Devinsky et al., 2018). These changes result in abnormal network formation, changes in cell excitability, and ultimately spontaneous seizures. To date, numerous changes in protein expression levels, expression sites, molecular structure and function have been identified during the process of epilepsy and even after epilepsy development (Pitkänen and Lukasiuk, 2009). Previous studies have reported significantly increased Beclin1 expression in PTZ-treated epileptic rats (Zhu et al., 2016) and KA-induced epilepsy models (Cao et al., 2020). Consistent with these results, we also detected increased Beclin1 levels in both KA-induced and PTZ-kindled epileptic mice. Notably, we first confirmed elevated Beclin1 protein levels in the cortices of patients with TLE.

Autophagy is characterized by the engulfment of cellular components into double-membrane or multiple-membrane cytoplasmic vesicles called autophagosomes that form from a membranous structure called the phagophore. Autophagosomes ultimately fuse with lysosomes, forming autolysosomes, which ultimately degrade the engulfed proteins or organelles. This whole process is called autophagy flux (Ge et al., 2022). An increase in the level of the LC3-II isoform potentially indicates reduced autophagy flux (Klionsky et al., 2021). Considering that Beclin1 is a key molecule involved in autophagy, we further evaluated autophagy flux in epilepsy and observed that the ratio of LC3-II to LC3-I was decreased, while p62 accumulated in epileptic mice, these findings are consistent with previous research (Li et al., 2018) suggesting an activation in autophagy flux rather than the impairment of autophagy.

We next investigated whether the elevated level of Beclin1 correlated with epileptic seizures in mice with a heterozygous disruption of *beclin1*. Previous studies have observed that autophagy is inhibited in mice with incomplete knockout of *beclin1* (*beclin1*^±^ mice) (Sun et al., 2018). Several investigations have reported that inhibiting autophagy reduces the susceptibility to epilepsy in KA-induced or pilocarpine-induced epileptic mice (Jeong et al., 2015; Ying et al., 2020), implying that the suppression of autophagy is sufficient to inhibit epilepsy. We measured autophagy flux to determine the level of autophagy in *beclin1*^±^ mice. In *beclin1*^±^ mice, autophagy flux was lower than that in WT mice, indicating that incomplete knockout of *beclin1* inhibited autophagy. An autophagy inducer (rapamycin) and inhibitor (3-MA) were used to observe the corresponding changes in autophagy flux. Our results revealed that rapamycin altered autophagy flux in *beclin1*^±^ mice, while 3-MA had little effect.

Imbalances between excitatory and inhibitory signals have been suggested to cause epilepsy. AEDs that are currently used to stop epileptic seizures act mainly by inhibiting neuronal excitability, blocking ion channels and inhibiting synaptic neurotransmitter release (Kwon et al., 2019). Studies have provided insights into the genes involved in synaptic transmission or the regulation of synaptic transmission (Pitkänen and Lukasiuk, 2011). We next focused on the effects of Beclin1 on synaptic transmission. Our results first revealed that both the frequency and amplitude of mEPSCs were decreased in *beclin1*^±^ mice. As epileptic activity is caused by an imbalance in excitatory and inhibitory transmission (Rubenstein and Merzenich, 2003), the unaffected mIPSCs in the *beclin1*^±^ group in our study suggest that Beclin1 leads to a hypoexcitable state by decreasing excitatory transmission.

In a patch-clamp experiment, incomplete knockout of *beclin1* was shown to affect the NMDAR-mediated current but not the AMPAR-mediated current. According to previous studies, autophagy exerts a protective effect on the process of NMDAR-mediated excitotoxicity (Pérez-Carrión et al., 2012; Pérez-Carrión and Ceña, 2013). We assessed neuronal PPRs to further explore whether the NMDAR-mediated current was affected by presynaptic or postsynaptic changes. The results of PPR and colocalization analyses indicated that incomplete knockout of *beclin1* affected synaptic transmission mainly *via* postsynaptic effects. An experiment on long-term social isolation found that, long-term social isolation can reduce the protein level of Beclin1, inhibit autophagy, and lead to postsynaptic dysfunction, impairment of spatial memory and cognitive function (Wang et al., 2019). Therefore, in our study, the current changes may be related to the inhibition of autophagy, which is in turn related to Beclin1. Overall, our study suggests that Beclin1 may alter excitatory synaptic transmission at the postsynaptic site, eventually resulting in epilepsy.

Dendritic spines are small, thin, specialized protrusions from neuronal dendrites that are primarily localized in excitatory synapses (Chidambaram et al., 2019) and usually receive and integrate most excitatory synaptic inputs from the mammalian cortex and hippocampus and directly affect neuronal excitability and seizures under pathological conditions (Wong and Guo, 2013). Since dendritic spines constitute the most important parts of excitatory synapses, their morphology and density play crucial roles in synaptic plasticity (Chidambaram et al., 2019). We found that the dendritic spine density was decreased in the neurons of *beclin1*^±^ mice, indicating that incomplete deletion of *beclin1* alters synaptic transmission by inhibiting synapse formation. However, we observed that this defect was not significantly reversed by the autophagy inducer rapamycin *in vitro*. More *in vivo* studies are needed to further verify this finding.

Autophagy is a catabolic process that liberates free amino acids through protein degradation. Impaired autophagy might disturb the homeostasis of neurotransmitters that are implicated in brain physiology and pathophysiology (Bejarano and Rodríguez-Navarro, 2015). Based on this justification, the altered excitatory synaptic transmission in *beclin1*^±^ mice in this study may be explained by the alterations in autophagy caused by Beclin1 deficiency. However, further exploration is needed to confirm this conjecture in the future.

## Conclusion

In summary, we report a previously unrecognized but important role of Beclin1 in epilepsy. Our results reveal that Beclin1 expression is increased in brain tissues from patients with TLE. Additionally, heterozygous disruption of *beclin1* significantly decreases seizure activity and may be involved in altered excitatory synaptic transmission caused by abnormal dendritic spine formation. These findings contribute to our understanding of the biological roles of Beclin1 at synapses. Moreover, this study provides insights into the development of an alternative approach for epilepsy treatment by identifying a novel therapeutic target, Beclin1.

## Data Availability Statement

The original contributions presented in this study are included in the article/Supplementary Material, further inquiries can be directed to the corresponding authors.

## Ethics Statement

The studies involving human participants were reviewed and approved by the Ethics Committee of the First Affiliated Hospital of Chongqing Medical University. The patients/participants were informed to participate in this study. The animal study was reviewed and approved by the Ethics Committee of Chongqing Medical University.

## Author Contributions

MY, PL, XT, CC, XW, and FX conceived the project and designed the experiments. MY, PL, WJ, HG, HC, YC, YG, YxG, MH, JW, XJ, ZZ, and XX performed the experiments. MY, PL, XT, CC, and FX analyzed the data. MY, XT, and XW wrote the manuscript. All the authors revised and approved the final version of the manuscript.

## Conflict of Interest

The authors declare that the research was conducted in the absence of any commercial or financial relationships that could be construed as a potential conflict of interest. The handling editor YJ declared a past co-authorship with the author XW.

## Publisher’s Note

All claims expressed in this article are solely those of the authors and do not necessarily represent those of their affiliated organizations, or those of the publisher, the editors and the reviewers. Any product that may be evaluated in this article, or claim that may be made by its manufacturer, is not guaranteed or endorsed by the publisher.

## Supplementary Material

Supplementary Figure 1 Knockdown of *beclin1* altered the intrinsic excitability of neurons. **(A,B)** Beclin1 protein levels in the cortex and hippocampus of brain tissues from wild-type (WT) and *beclin1*^±^ mice (cortex: *n* = 5 mice per group; hippocampus: *n* = 4 mice per group). **(C)** Paradigm used to record passive excitability in the excitatory neurons. **(D)** Representative traces of sAPs. **(E)** Resting membrane potential of the examined neurons from the two groups (*n* = 6 per group). **(F)** Injected currents used to induce the first spikes (*n* = 6 per group). **(G)** Summary of the number of APs induced by the injected currents (*n* = 6 per group). Data are presented as the means ± SEM. ^**^*p* < 0.01. Student’s *t*-tests were performed.

## References

[B1] Ashkenazi A. Bento C. F. Ricketts T. Vicinanza M. Siddiqi F. Pavel M. (2017). Polyglutamine tracts regulate beclin 1-dependent autophagy. *Nature* 545 108–111. 10.1038/nature22078 28445460 PMC5420314

[B2] Bejarano E. Rodríguez-Navarro J. A. (2015). Autophagy and amino acid metabolism in the brain: implications for epilepsy. *Amino Acids* 47 2113–2126. 10.1007/s00726-014-1822-z 25145921

[B3] Cao J. Tang C. Gao M. Rui Y. Zhang J. Wang L. (2020). Hyperoside alleviates epilepsy-induced neuronal damage by enhancing antioxidant levels and reducing autophagy. *J. Ethnopharmacol.* 257:112884. 10.1016/j.jep.2020.112884 32311482

[B4] Chidambaram S. B. Rathipriya A. G. Bolla S. R. Bhat A. Ray B. Mahalakshmi A. M. (2019). Dendritic spines: revisiting the physiological role. *Prog. Neuropsychopharmacol. Biol. Psychiatry* 92 161–193. 10.1016/j.pnpbp.2019.01.005 30654089

[B5] Criado O. Aguado C. Gayarre J. Duran-Trio L. Garcia-Cabrero A. M. Vernia S. (2012). Lafora bodies and neurological defects in malin-deficient mice correlate with impaired autophagy. *Hum. Mol. Genet.* 21 1521–1533. 10.1093/hmg/ddr590 22186026

[B6] Devinsky O. Vezzani A. O’Brien T. J. Jette N. Scheffer I. E. de Curtis M. (2018). Epilepsy. *Nat. Rev. Dis. Primers* 4:18024. 10.1038/nrdp.2018.24 29722352

[B7] Egan D. Kim J. Shaw R. J. Guan K. L. (2011). The autophagy initiating kinase ULK1 is regulated *via* opposing phosphorylation by AMPK and mTOR. *Autophagy* 7 643–644. 10.4161/auto.7.6.15123 21460621 PMC3359466

[B8] Engel J. Jr. (2001). A proposed diagnostic scheme for people with epileptic seizures and with epilepsy: report of the ILAE task force on classification and terminology. *Epilepsia* 42 796–803. 10.1046/j.1528-1157.2001.10401.x 11422340

[B9] Falconer M. A. Taylor D. C. (1968). Surgical treatment of drug-resistant epilepsy due to mesial temporal sclerosis. Etiology and significance. *Arch. Neurol.* 19 353–361. 10.1001/archneur.1968.00480040019001 5677186

[B10] Fisher R. S. Acevedo C. Arzimanoglou A. Bogacz A. Cross J. H. Elger C. E. (2014). ILAE official report: a practical clinical definition of epilepsy. *Epilepsia* 55 475–482. 10.1111/epi.12550 24730690

[B11] Ge P. Lei Z. Yu Y. Lu Z. Qiang L. Chai Q. (2022). *M. tuberculosis* PknG manipulates host autophagy flux to promote pathogen intracellular survival. *Autophagy* 18 576–594. 10.1080/15548627.2021.1938912 34092182 PMC9037497

[B12] Guo Y. Chen Y. Yang M. Xu X. Lin Z. Ma J. (2020). A rare KIF1A missense mutation enhances synaptic function and increases seizure activity. *Front. Genet.* 11:61. 10.3389/fgene.2020.00061 32174959 PMC7056823

[B13] Jeong K. H. Jung U. J. Kim S. R. (2015). Naringin attenuates autophagic stress and neuroinflammation in kainic acid-treated hippocampus *in vivo*. *Evid. Based Complement. Alternat. Med.* 2015:354326. 10.1155/2015/354326 26124853 PMC4466392

[B14] Klionsky D. J. Abdel-Aziz A. K. Abdelfatah S. Abdellatif M. Abdoli A. Abel S. (2021). Guidelines for the use and interpretation of assays for monitoring autophagy (4th edition)(1). *Autophagy* 17 1–382. 10.1080/15548627.2020.1797280 33634751 PMC7996087

[B15] Kwon J. Y. Jeon M. T. Jung U. J. Kim D. W. Moon G. J. Kim S. R. (2019). Perspective: therapeutic potential of flavonoids as alternative medicines in epilepsy. *Adv. Nutr.* 10 778–790. 10.1093/advances/nmz047 31111873 PMC6743823

[B16] Levine B. Kroemer G. (2019). Biological functions of autophagy genes: a disease perspective. *Cell* 176 11–42. 10.1016/j.cell.2018.09.048 30633901 PMC6347410

[B17] Li J. Mi X. Chen L. Jiang G. Wang N. Zhang Y. (2016). Dock3 participate in epileptogenesis through rac1 pathway in animal models. *Mol. Neurobiol.* 53 2715–2725. 10.1007/s12035-015-9406-9 26319681

[B18] Li Q. Han Y. Du J. Jin H. Zhang J. Niu M. (2018). Alterations of apoptosis and autophagy in developing brain of rats with epilepsy: changes in LC3, P62, Beclin-1 and Bcl-2 levels. *Neurosci. Res.* 130 47–55. 10.1016/j.neures.2017.08.004 28807642

[B19] Liang C. Feng P. Ku B. Dotan I. Canaani D. Oh B. H. (2006). Autophagic and tumour suppressor activity of a novel Beclin1-binding protein UVRAG. *Nat. Cell Biol.* 8 688–699. 16799551 10.1038/ncb1426

[B20] Liang X. H. Jackson S. Seaman M. Brown K. Kempkes B. Hibshoosh H. (1999). Induction of autophagy and inhibition of tumorigenesis by beclin 1. *Nature* 402 672–676. 10.1038/45257 10604474

[B21] Liang X. H. Kleeman L. K. Jiang H. H. Gordon G. Goldman J. E. Berry G. (1998). Protection against fatal Sindbis virus encephalitis by beclin, a novel Bcl-2-interacting protein. *J. Virol.* 72 8586–8596. 9765397 10.1128/jvi.72.11.8586-8596.1998 PMC110269

[B22] Liu X. Wang B. (2019). Heterozygous disruption of beclin 1 alleviates zinc oxide nanoparticles-induced disturbance of cholesterol biosynthesis in mouse liver. *Int. J. Nanomed.* 14 9865–9875. 10.2147/ijn.s224179 31849474 PMC6913297

[B23] McDaniel S. S. Rensing N. R. Thio L. L. Yamada K. A. Wong M. (2011). The ketogenic diet inhibits the mammalian target of rapamycin (mTOR) pathway. *Epilepsia* 52 e7–e11. 10.1111/j.1528-1167.2011.02981.x 21371020 PMC3076631

[B24] Mizushima N. Yoshimori T. (2007). How to interpret LC3 immunoblotting. *Autophagy* 3 542–545. 10.4161/auto.4600 17611390

[B25] Pérez-Carrión M. D. Ceña V. (2013). Knocking down HMGB1 using dendrimer-delivered siRNA unveils its key role in NMDA-induced autophagy in rat cortical neurons. *Pharm. Res.* 30 2584–2595. 10.1007/s11095-013-1049-9 23604926

[B26] Pérez-Carrión M. D. Pérez-Martínez F. C. Merino S. Sánchez-Verdú P. Martínez-Hernández J. Luján R. (2012). Dendrimer-mediated siRNA delivery knocks down Beclin 1 and potentiates NMDA-mediated toxicity in rat cortical neurons. *J. Neurochem.* 120 259–268. 10.1111/j.1471-4159.2011.07556.x 22035151

[B27] Pitkänen A. Lukasiuk K. (2009). Molecular and cellular basis of epileptogenesis in symptomatic epilepsy. *Epilepsy Behav.* 14(Suppl. 1) 16–25. 10.1016/j.yebeh.2008.09.023 18835369

[B28] Pitkänen A. Lukasiuk K. (2011). Mechanisms of epileptogenesis and potential treatment targets. *Lancet Neurol.* 10 173–186. 10.1016/s1474-4422(10)70310-0 21256455

[B29] Racine R. J. (1972). Modification of seizure activity by electrical stimulation. II. Motor seizure. *Electroencephalogr. Clin. Neurophysiol.* 32 281–294. 10.1016/0013-4694(72)90177-0 4110397

[B30] Rubenstein J. L. Merzenich M. M. (2003). Model of autism: increased ratio of excitation/inhibition in key neural systems. *Genes Brain Behav.* 2 255–267. 10.1034/j.1601-183x.2003.00037.x 14606691 PMC6748642

[B31] Sun Y. Yao X. Zhang Q. J. Zhu M. Liu Z. P. Ci B. (2018). Beclin-1-dependent autophagy protects the heart during sepsis. *Circulation* 138 2247–2262. 10.1161/circulationaha.117.032821 29853517 PMC6274625

[B32] Wang B. Wu Q. Lei L. Sun H. Michael N. Zhang X. (2019). Long-term social isolation inhibits autophagy activation, induces postsynaptic dysfunctions and impairs spatial memory. *Exp. Neurol.* 311 213–224. 10.1016/j.expneurol.2018.09.009 30219732

[B33] Wong M. (2013). Cleaning up epilepsy and neurodegeneration: the role of autophagy in epileptogenesis. *Epilepsy Curr.* 13 177–178. 10.5698/1535-7597-13.4.177 24009482 PMC3763603

[B34] Wong M. Guo D. (2013). Dendritic spine pathology in epilepsy: cause or consequence? *Neuroscience* 251 141–150. 10.1016/j.neuroscience.2012.03.048 22522469

[B35] Yang Y. Tian X. Xu D. Zheng F. Lu X. Zhang Y. (2018). GPR40 modulates epileptic seizure and NMDA receptor function. *Sci. Adv.* 4:eaau2357. 10.1126/sciadv.aau2357 30345361 PMC6192686

[B36] Ying C. Ying L. Yanxia L. Le W. Lili C. (2020). High mobility group box 1 antibody represses autophagy and alleviates hippocampus damage in pilocarpine-induced mouse epilepsy model. *Acta Histochem.* 122:151485. 10.1016/j.acthis.2019.151485 31870503

[B37] Zavala-Tecuapetla C. Cuellar-Herrera M. Luna-Munguia H. (2020). Insights into potential targets for therapeutic intervention in epilepsy. *Int. J. Mol. Sci.* 21:8573. 10.3390/ijms21228573 33202963 PMC7697405

[B38] Zhang H. Tian X. Lu X. Xu D. Guo Y. Dong Z. (2019). TMEM25 modulates neuronal excitability and NMDA receptor subunit NR2B degradation. *J. Clin. Invest.* 129 3864–3876. 10.1172/jci122599 31424425 PMC6715386

[B39] Zhu X. Shen K. Bai Y. Zhang A. Xia Z. Chao J. (2016). NADPH oxidase activation is required for pentylenetetrazole kindling-induced hippocampal autophagy. *Free Radic. Biol. Med.* 94 230–242. 10.1016/j.freeradbiomed.2016.03.004 26969791

