# Supplementary figures and images for: Beclin1 Deficiency Suppresses Epileptic Seizures
Source: Front Mol Neurosci. 2022 Jul 22;15:807671. doi: 10.3389/fnmol.2022.807671 (PMC9354790; doi:10.3389/fnmol.2022.807671)

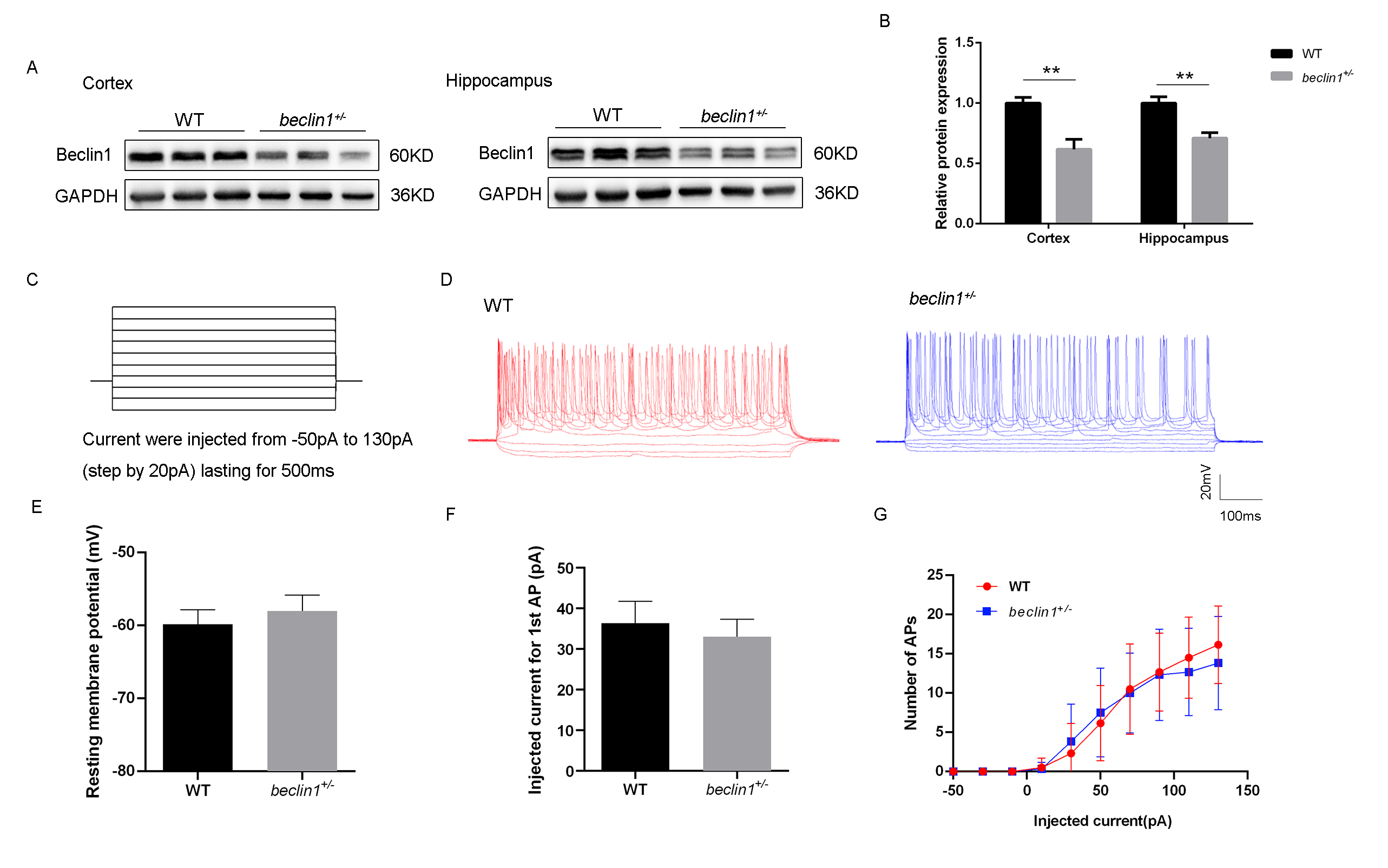

Supplement: Supplementary Figure 1 — Knockdown of beclin1 altered the intrinsic excitability of neurons. (A,B) Beclin1 protein levels in the cortex and hippocampus of brain tissues from wild-type (WT) and beclin1± mice (cortex: n = 5 mice per group; hippocampus: n = 4 mice per group). (C) Paradigm used to record passive excitability in the excitatory neurons. (D) Representative traces of sAPs. (E) Resting membrane potential of the examined neurons from the two groups (n = 6 per group). (F) Injected currents used to induce the first spikes (n = 6 per group). (G) Summary of the number of APs induced by the injected currents (n = 6 per group). Data are presented as the means ± SEM. **p < 0.01. Student’s t-tests were performed. [file Image_1.TIF]
